# Effects of and Prospects for the Hierarchical Medical Policy in Beijing, China

**DOI:** 10.3390/healthcare11081067

**Published:** 2023-04-08

**Authors:** Yongchuang Gao, Yuangeng Guo, Jianwei Deng

**Affiliations:** 1 School of Labor and Human Resources, Renmin University of China, 59 Zhongguancun Street, Beijing 100872, China; 2 School of Economics and Management, Tsinghua University, 30 Shuangqing Street, Beijing 100084, China; 3 School of Management and Economics, Beijing Institute of Technology, 5 Zhongguancun South Street, Beijing 100081, China; 4 Sustainable Development Research Institute for Economy and Society of Beijing, 5 Zhongguancun South Street, Beijing 100081, China

**Keywords:** hierarchical medical policy, workload, medical seeking behavior, hospital management, government data

## Abstract

Hierarchical medical policies are widely used worldwide to reduce healthcare costs, rationalize the use of healthcare resources, and improve accessibility and fairness of healthcare services. However, few case studies have evaluated the effects and prospects of such policies. Medical reform efforts in China have distinct goals and characteristics. Therefore, we investigated the effects of a hierarchical medical policy in Beijing and assessed the future potential of the policy to yield insights for other countries, especially developing countries. Different methods were used to analyze multidimensional data from official statistics, a questionnaire survey of 595 healthcare workers from 8 representative public hospitals in Beijing, a questionnaire survey of 536 patients, and 8 semi-structured interview records. The hierarchical medical policy had strong positive effects on improving access to healthcare services, balancing the workload of healthcare workers in various levels of public hospitals, and improving the management of public hospitals. The remaining obstacles include severe job stress among healthcare workers, the high cost of some healthcare services, and the need for improvement in the development level and service capacity of primary hospitals. This study provides useful policy recommendations regarding the implementation and extension of the hierarchical medical policy, including the need for governments to improve the hospital assessment system and for hospitals to actively participate in developing medical partnerships.

## 1. Introduction

Reducing healthcare costs, rationalizing the use of healthcare resources, and improving access to and fairness of healthcare services are major challenges in healthcare reform efforts in many countries [1,2,3,4,5]. In China, the demand for healthcare services is vast, and the distribution of healthcare resources is inequitable [6,7]. Therefore, the Chinese government started a new round of nationwide comprehensive healthcare reform in 2009; the reform’s goal was for everyone to enjoy basic medical and health care and improve the health of the whole population. Ten years later, because of the unique model of policy implementation (policy pilot) and the propagable reform experience, researchers around the world are evaluating these reforms [8,9,10].

Hierarchical medical policy means that hospitals at different levels provide different medical services, and the government and hospitals should guide different patients to appropriate hospitals; it is a trend in global healthcare and a signpost of healthcare reform in China [11]. Formally implemented by the central government of China in 2015, reforms have mainly focused on common and chronic diseases and have been implemented in 94.7% of cities at the prefectural level or higher in China [12,13]. Chinese public healthcare institutions can be classified as tertiary hospitals, secondary hospitals, primary hospitals [14], and other healthcare centers. If patients want to access medical care, they need to go through a series of activities, including registration, queuing, visiting the doctor, outpatient or inpatient treatment, and return visits. The essential goals of the hierarchical medical policy are to define the functional orientation of the diagnostic and treatment services offered by these different healthcare institutions, to encourage patients to receive initial diagnoses and treatment in lower-level healthcare institutions and to facilitate patient transfers between the different levels of hospitals. The policy objectives specify that tertiary hospitals should mainly provide diagnostic and treatment services for severe and complex diseases. Secondary hospitals are to mainly receive patients during convalescence after acute disease and after operations, as well as patients with stable severe diseases transferred from tertiary hospitals. Primary hospitals and basic healthcare centers are to provide treatment, rehabilitation, and nursing services for patients with chronic diseases, convalescing patients, geriatric patients, and patients with advanced cancer with definitive diagnoses and stable disease [12].

As the capital of China, Beijing is the pioneer and benchmark for the hierarchical medical policy and has explored reform with local characteristics. To ensure the progress and effectiveness of hierarchical medical reform, the Beijing municipal government formulated explicit, detailed implementation plans and annual key work than other pilot cities. In the first stage (from 2016 to 2017), the focus of reform was to increase resource inputs for primary hospitals and encourage patients to seek initial treatment at primary hospitals, thus responding to deficiencies in services and staffing at primary hospitals in Beijing [15]. In the second stage (from 2018 to 2020), the key tasks of reform are to further clarify the functional orientation of healthcare institutions at all levels and accelerate the implementation of supporting policies, e.g., family doctor policy, medical partnership policy, and an electronic diagnosis and treatment platform [16]. Although Beijing has accumulated considerable experience in implementing a hierarchical medical policy, new challenges require timely evaluation and solutions.

However, there are some gaps between the present research and reform progress and practical needs. Studies have investigated the effect of hierarchical medical policy on the medical treatment behavior of certain groups [17]. Some studies used big data to guide patients to receive initial diagnoses and treatment at primary hospitals [18]. Other work examined the optimization of the spatial layout of urban healthcare centers and the development of an efficient medical alliance [19]. Another study used a hesitant fuzzy linguistic analytic network to assess hierarchical medical policy proposals [20]. Nevertheless, existing evidence on the effects of hierarchical medical policy is inadequate, and reform of patient willingness and behavior remains a primary objective. Therefore, a comprehensive study of the effects of hierarchical medical policy on hospitals at all levels, healthcare workers, patients, and healthcare services at this critical stage of reform would not only enrich our understanding of the hierarchical medical policy but could also guide future reform of the hierarchical medical policy in Beijing and nationwide. More importantly, governments and researchers are increasingly suggesting that China has lessons for the world in areas such as public health, especially for developing countries [21,22]. This study comprehensively evaluated statistical, questionnaire, and interview data to determine the present and potential effects of a hierarchical medical policy in Beijing, thereby providing guidance for developed and developing countries seeking to reduce healthcare costs, rationalize the use of healthcare resources, and improve accessibility to and fairness of healthcare services.

## 2. Data and Methods

### 2.1. Data Sources

The data for this study are comprehensive and diverse and mainly include official statistics, questionnaire data, and interview data.

#### 2.1.1. Official Statistics

We collected official statistical data (e.g., annual total consultations and discharges, consultations per doctor per day) from the websites of the Beijing Municipal Health Commission Information Center (http://www.phic.org.cn/tjsj/wstjjb/, accessed on 10 November 2022.) and the Beijing Municipal Bureau of Statistics (http://www.bjstats.gov.cn/tjsj/, accessed on 10 November 2022.), mainly for the period from 2009 to 2018. The data collected from 2009 to 2015 can better be compared with the data changes after the implementation of the policy to better reveal the data changes and analyze the effect of the policy.

#### 2.1.2. Questionnaire Data

The questionnaire data were collected from a 2-wave questionnaire survey. Firstly, after obtaining ethical approval (No. KYX2016007) and informed consent, a cross-sectional questionnaire survey was distributed to 595 healthcare workers (response rate, 79.8%, which can obtain reliable data results) in 8 representative public healthcare institutions (including tertiary hospitals, secondary hospitals, primary hospitals, and basic healthcare centers) in Beijing from October 2019 to February 2020. In order to better evaluate the effect of the policy, two public hospitals at each level were selected in this study through random stratified sampling, representing all different levels of hospitals and different regional levels. Considering the scale of the hospitals and the number of healthcare workers, and in order to guarantee data integrity and objectivity, we used employee identification numbers to randomly select 5% to 10% of healthcare workers from each studied hospital through random stratified sampling. The main variables of interest were job satisfaction, job stress, distributive justice, and work performance of healthcare workers after the implementation of a hierarchical medical policy.

The hierarchical medical policy affects both healthcare workers and patients. We conducted an online questionnaire survey of Beijing residents in February 2020, which yielded 536 valid questionnaires (the sample size can ensure this study obtains reliable data results). The questionnaire included questions on policy awareness, attitudes and behaviors of policy participation, policy satisfaction, and factors influencing policy implementation. To identify appropriate respondents, the beginning of the questionnaire included the questions, “Have you lived in Beijing for the past 3 years?” and “Have you been to a Beijing public hospital (or basic healthcare center) in the past year?”. Participants who answered negatively to either question were excluded. After these two questions, the survey can make sure that the participants have more experience, participation, and understanding of hierarchical medical policy.

#### 2.1.3. Interview Data

We used a purposeful sampling method to recruit 8 participants for individual interviews during the period from October to December 2019. The 8 participants were administrative staff from representative public healthcare centers in Beijing, 1 per hospital, 2 from each level hospital, including different level hospitals; they had different degrees of understanding and participation in the hierarchical medical policy and were expected to provide useful and specific information. The interviews were semi-structured and conducted by telephone. The duration of most of the interviews was 20 to 30 min, and information was collected on the effects, challenges, and trends in hierarchical medical policy from the perspective of hospital management.

### 2.2. Measures

We used a 6-item scale to measure the job satisfaction of healthcare workers [23], which had high reliability in our research (α = 0.755). For example, the item “I am very content with my job” evaluates job satisfaction. The scale uses a 5-point Likert scale to evaluate the job satisfaction of respondents (1 = strongly disagree; 5 = strongly agree).

We used the 11-item Challenge and Hindrance-related Self-Reported Stress (C-HSS) scale to measure the job stress of healthcare workers [24], which had high reliability in our research (α = 0.910). For example, the item “The volume of work that must be accomplished in the allotted time” evaluates challenge stress, while the item “The amount of red tape I need to go through to get my job done” evaluates hindrance stress. The C-HSS scale uses a 5-point Likert scale to evaluate the challenge stress and hindrance stress of respondents (1 = no stress; 5 = great stress).

Distributive justice is an important dimension of organizational justice and was measured with a 5-item scale assessing the fairness of different work outcomes [25]. It had high reliability in our research (α = 0.932). For example, the item “I think that my level of pay is fair” evaluates distributive justice, including pay level, work schedule, workload, and job responsibility [25]. The scale uses a 5-point Likert scale to evaluate distributive justice (1 = strongly disagree; 5 = strongly agree).

To assess the work performance of healthcare workers, we used a 4-item scale [26], which had high reliability in our research (α = 0.907). For example, the item “Quality of your performance” evaluates work performance. The scale uses a 5-point Likert scale to evaluate the work performance of respondents (1 = strongly disagree; 5 = strongly agree).

We developed a 4-part questionnaire to measure patient awareness, participation, and satisfaction regarding the hierarchical medical policy (α = 0.774). In Part 1, the item “I understand the hierarchical medical policy very well” (1 = strongly disagree; 5 = strongly agree) was used to investigate patient awareness of the hierarchical medical policy. In Part 2, respondents were asked to select their preferred healthcare institution—tertiary hospital, secondary hospital, primary hospital, or basic healthcare center—for 9 disease categories (e.g., acute disease, chronic disease, severe disease). In Part 3, respondents were asked to rank the importance of factors affecting their choice of healthcare facility (1 = very unimportant; 5 = very important). In Part 4, we investigated the overall satisfaction of respondents with the hierarchical medical policy and with specific policy measures. A 5-point Likert scale was used to evaluate respondent satisfaction (1 = very dissatisfied; 5 = very satisfied).

### 2.3. Methods

Different methods were used to process the multidimensional data. For quantitative analysis, after all the information was collected from the statistical database and healthcare worker and patient questionnaires, SPSS 24.0 was used for descriptive analysis and analysis of variance.

In addition to quantitative analysis, we used thematic content analysis to conduct a simple qualitative analysis. When contacting participants, we explained the purpose and general content of the interview and informed them that the interviews would be recorded. The interview was started only after obtaining participant consent. During the interview, all the interview content was digitally recorded and transcribed verbatim, and translated into English for analysis; the main points of the interview were noted. Later, one student assistant transcribed all the interviews, and another student assistant verified the accuracy of the transcripts. Careful and repeated reading of the transcribed texts helped the researchers identify the quality of the content. The data were coded manually, and relevant themes were inductively and deductively identified and categorized based on similarities and differences. After that, the interview data verified the results of the quantitative analysis.

## 3. Results

### 3.1. Workload of Healthcare Workers

#### 3.1.1. Annual Total Consultations and Discharges

The statistical data showed that (1) during 2009–2016, although the annual total number of consultations in tertiary hospitals increased every year, the rate of increase slowed after the implementation of the hierarchical medical policy, (2) unlike tertiary hospitals, the annual total number of consultations in primary hospitals exhibited an upward trend overall, and the growth rate was significantly faster after implementation, (3) the annual total number of consultations in secondary hospitals was relatively stable, and (4) from 2016, after implementation, the annual total number of discharges from hospital increased for tertiary hospitals and remained stable for other public hospitals (Table 1).

#### 3.1.2. Consultations Per Doctor Per Day

The analysis showed that the number of consultations per doctor per day (1) was distinctly higher in primary hospitals than in other public hospitals, (2) distinctly decreased, to the level of 2010, in tertiary hospitals after implementation of the hierarchical medical policy, and (3) declined modestly in secondary hospitals after implementation (Table 2).

#### 3.1.3. Inpatient Bed Days Per Doctor Per Day

The statistical results showed that after implementation of the hierarchical medical policy, inpatient bed days per doctor per day (1) did not significantly change in tertiary hospitals, although the annual total number of discharged patients increased obviously, which suggests an increase in work intensity, and (2) decreased obviously in primary hospitals but did not change in secondary hospitals (Table 3).

#### 3.1.4. Number of Referrals

We reviewed the relevant data on the number of referrals since the implementation of the hierarchical medical policy, including upward referrals (from low-level hospitals to high-level hospitals) and downward referrals (from high-level hospitals to low-level hospitals). Since the implementation of the hierarchical medical policy in 2015, two-way referrals have been common between Beijing hospitals of different levels. The number of referrals exceeded 200,000 in 2015, increased to 260,000 at the end of 2016, and are continuing to increase.

### 3.2. Subjective Perception of Healthcare Workers

#### 3.2.1. Job Satisfaction

The average values for the six items on job satisfaction were very high (range, 3.43–3.98). The scores are equal to or higher than 3.43, indicating that healthcare workers had very high job satisfaction. Job satisfaction of healthcare workers at the different levels of hospitals differed obviously, however. Job satisfaction was higher among healthcare workers in high-level hospitals and was distinctly higher in tertiary hospitals than in community healthcare centers (Table 4 and Table 5).

#### 3.2.2. Job Stress

The average value was higher for challenge stress than for hindrance stress. The average value for the six items on challenge stress was very high (range, 3.38–3.47), and the average value for the six items on hindrance stress was low (range, 2.43–3.09). The scores of challenge stress are equal to or higher than 3.30; hindrance stress are equal to or higher than 2.79 in different hospitals, which means high job stress. Job stress among healthcare workers obviously differed in relation to the hospital level, challenge stress did not obviously differ in relation to the hospital level, and hindrance stress was highest in community healthcare centers and lowest at secondary hospitals (Table 4 and Table 5).

#### 3.2.3. Distributive Justice

The average values for the five items on distributive justice were very high (range, 3.31–3.61). The scores are equal to or higher than 3.31, indicating that healthcare workers had very high distributive justice. Distributive justice among healthcare workers distinctly differed in relation to the hospital level: it was highest in secondary hospitals and lowest in primary hospitals, but the difference in values was not large (Table 4 and Table 5).

#### 3.2.4. Work Performance

The average values for the four items on work performance were very high (range, 4.00–4.05). The scores are equal to or higher than 4.00, which means that healthcare workers had very high work performance. The work performance of healthcare workers obviously differed in relation to the hospital level: work performance was highest for healthcare workers in secondary hospitals and lowest for community healthcare centers (Table 4 and Table 5).

### 3.3. Patient Awareness, Participation, and Satisfaction Regarding the Hierarchical Medical Policy

#### 3.3.1. Awareness

Significantly more respondents understood the hierarchical medical policy than did not. The percentage answering “more agree” was the highest, indicating that most respondents had some understanding of the hierarchical medical policy (Table 6).

#### 3.3.2. Medical Treatment Sought by Disease Type

For common diseases, most respondents chose basic healthcare centers and primary hospitals. For acute, chronic, and severe diseases, most people chose tertiary and secondary hospitals and most of those preferred tertiary hospitals. For incurable diseases, most people chose tertiary hospitals. For disease convalescence, the numbers of people seeking care were more balanced in relation to hospital type, which contrasted with their preferences for the other disease categories. In general, most people preferred to be treated in tertiary hospitals when ill. The difference in the number of people who preferred primary hospitals and community health centers was very small (Table 7).

#### 3.3.3. Factors Affecting Medical Behavior

The average values for the eight factors for medical behavior were very high (range, 3.92–4.48). The scores of each factor are equal to or higher than 3.92, which means that the importance of them is very high. Respondents reported that “severe condition” and “the level of the physician’s diagnosis and treatment” were the most important factors affecting the hospital type they selected (Table 8).

#### 3.3.4. Satisfaction with the Hierarchical Medical Policy

The average values for the eight measures were very high (range, 3.55–3.77), and the scores of each item were equal to or higher than 3.55, indicating that many people had very high satisfaction with the measures included in the hierarchical medical policy. The item “Information collection/retrieval is gradually improving, making it more convenient for patients to see a doctor” is closely related to the increasingly sophisticated electronic systems used in hospitals and received the highest score. Digitization of medical information makes medical treatment more convenient for patients. However, the score for the item “signing rate of family doctors increased, and the number of general practitioners increased” was relatively low; the signing rate represents the percentage of the public that has family doctors, and the score suggests that people were not satisfied with this measure. In general, significantly more people were satisfied with the hierarchical medical policy than not satisfied (Table 9 and Table 10).

### 3.4. Public Hospital Management

#### 3.4.1. Per Capita Cost of Outpatients and Inpatients

In the early stage of implementing the hierarchical medical policy, the per capita cost of outpatients in secondary hospitals continued to increase but started to decline in 2018. With implementation, the per capita cost of outpatients increased in tertiary hospitals and greatly increased in primary hospitals, the per capita cost of inpatients in primary hospitals increased rapidly and then obviously decreased, and the per capita cost of inpatients in secondary and tertiary hospitals increased year by year (Table 11).

#### 3.4.2. Drug Cost Ratio of Outpatients and Inpatients

Drug ratio refers to “drug income/total medical income”. In other words, it is the proportion of the cost of the medicine to the total cost of the patient’s visit to the doctor. With the implementation of the hierarchical medical policy, the drug cost ratio of outpatients and inpatients in tertiary and secondary hospitals significantly decreased year by year, the drug cost ratio of outpatients in primary hospitals first decreased and then increased dramatically, which is the only drug cost ratio index that rose during the reform process, but the drug cost ratio of inpatients obviously decreased and was the lowest among all hospitals levels. In addition, the drug cost ratio of inpatients in primary hospitals gradually decreased from the highest to the lowest of the hospital types (Table 12).

#### 3.4.3. Usage Rate of Prepared and Available Beds

The usage rate of prepared beds was significantly higher in secondary and tertiary hospitals than in primary hospitals. With the implementation of the hierarchical medical policy, the usage rate of prepared beds in tertiary hospitals increased over time. After 2016, the usage rate of prepared beds in secondary hospitals initially increased and then decreased, while the rate in primary hospitals initially decreased and then increased. Since implementation, the usage rate of available beds in tertiary hospitals has generally increased. Although the annual total number of discharged patients in tertiary hospitals increased in 2017, the usage rate of available beds decreased. From 2016, the usage rate of available beds initially increased and then decreased in secondary hospitals and initially decreased and then increased in primary hospitals (Table 13).

#### 3.4.4. Average Duration of Hospitalization for Discharged Patients

During the implementation of the hierarchical medical policy, the average duration of hospitalization decreased over time in tertiary hospitals and was significantly shorter than in other types of public hospitals. During implementation, the average duration was relatively stable in primary hospitals. Although the duration was longer than in other types of public hospitals, it decreased to two-thirds of the average duration in 2009. During implementation, the average duration of hospitalization initially decreased and then increased in secondary hospitals and initially increased and then decreased in primary hospitals (Table 14).

## 4. Discussion

To understand and predict the effects of the implementation of the hierarchical medical policy, we investigated and analyzed the workload of healthcare workers, the subjective perceptions of healthcare workers and patients, and changes in relevant indicators in all hospital types. Analysis of these multidimensional data yielded valuable and important findings.

### 4.1. Effects on Healthcare Workers

Healthcare reforms have substantial effects on, and are expected to inspire, healthcare workers [27]. Improving enthusiasm and satisfaction among healthcare workers improves patient satisfaction and quality of service, which leads to social benefits and promotes the economic growth of hospitals [28]. Therefore, one intended effect of the hierarchical medical policy is to have a positive effect on healthcare workers.

As expected, the hierarchical medical policy made workloads more balanced and reasonable for healthcare workers in hospitals. In the past, healthcare services in China were mainly provided by secondary and tertiary hospitals, and the healthcare role of lower-level hospitals was ignored [29]. The situation has improved since the implementation of the hierarchical medical policy. The annual total number of consultations has continuously decreased in tertiary hospitals but has greatly and progressively increased in primary hospitals. The number of consultations per doctor per day has decreased significantly in tertiary hospitals, from 11.94 (person-times) in 2015 to 9.74 in 2018, which was lower than in primary hospitals. In addition, the number of inpatient bed days per doctor per day was higher in tertiary hospitals than in other types of hospitals. Interviews yielded similar findings, for example, “In my hospital, many doctors work only part of the week in the outpatient department and most of the time in the inpatient department. This is a big difference between us and primary hospitals” (R 1, male administrator from a tertiary hospital). “According to the goals of hierarchical medical policy, tertiary hospitals are primarily positioned to provide inpatient service. Therefore, consultations per doctor per day in my hospital are declining and lower than in most secondary and primary hospitals” (R 2, female administrator from a tertiary hospital).

In our opinion, in addition to changes in patient health-seeking behavior, referral measures have led to more balanced and reasonable workloads for healthcare workers in all hospital types. We found that the number of two-way referrals in Beijing increased significantly since the implementation of the hierarchical medical policy. After implementation, tertiary hospitals are now more likely to treat inpatients with severe or incurable diseases, and low-level hospitals have greater responsibility for treating outpatients. These changes have reduced workloads for healthcare workers in large hospitals and have contributed to the goal of “primary consultation”.

However, although the hierarchical medical policy has improved workloads, healthcare workers continue to report severe job stress. Challenge stress is still high among healthcare workers in all types of hospitals, and especially in tertiary hospitals because the number of patients and their expectations for quality of care are rising [30,31]. Moreover, frequent incidents of medical violence and the large variety of work tasks increase challenge stress [32]. Fortunately, hindrance stress was low among healthcare workers at all hospital levels, particularly in secondary hospitals, which suggests good working relationships with supervisors and coworkers. The interviews were consistent with this interpretation. “The implementation of hierarchical medical policy has changed the work content of healthcare workers but hasn’t reduced workloads; they still face severe job stress” (R 2, female administrator from a tertiary hospital). “In my opinion, the job stress of healthcare workers mainly comes from the long working hours and heavy workload. In addition, misunderstandings with patients and medical violence also cause a lot of stress” (R 4, male administrator from a secondary hospital).

Interestingly, high job stress did not affect the perception of distributive justice, job satisfaction, or work performance of healthcare workers in any hospital type. Healthcare workers from primary hospitals and basic healthcare centers reported lower distributive justice, job satisfaction, and work performance than did those from higher-level hospitals. Thus, after the implementation of the hierarchical medical policy, the main problem faced by healthcare workers from lower-level facilities may not be job stress but low income, which could affect job satisfaction and work performance. To better promote the hierarchical medical policy, relevant departments need to consider gradually increasing the incomes of healthcare workers, which also responds to the advocacy of Mehran et al. [33]. At the same time, studies have shown that work stress, salary, and working environment affect the career choice of healthcare workers and even affect whether medical students will enter the medical field [34,35], which also reminds policymakers to consider these influencing factors. This was reflected in our interviews; “Implementation of hierarchical medical policy has not improved incomes of healthcare workers, and the mismatch between income and workload has reduced our job satisfaction. But it doesn’t affect our work performance because there are so many patients that we need to diagnose and treat every day. At the same time, we need to be responsible for the health and safety of patients” (R 5, male administrator from a primary hospital); “In our opinion, if the income of healthcare workers can be increased, it would help to improve their work performance and promote implementation of the hierarchical medical policy” (R 6, female administrator from a primary hospital).

### 4.2. Effects on Patients

Successful implementation of the hierarchical medical policy is closely related to patient awareness, participation, and satisfaction regarding such policy. After early policy publicity and implementation, most patients had some understanding of the policy, but 22.2% said they knew little or nothing about it. This indicates that the Beijing municipal government should increase public awareness and enrich publicity channels to increase policy coverage and penetration.

Patient participation in the hierarchical medical policy lessens the burdens of large hospitals and increases access to healthcare services. A previous study concluded that because primary hospitals lack doctors and medical facilities, an increasing number of urban residents want to be treated at large hospitals, regardless of the nature of their disease [29]. However, our study found that many patients adhered to the hierarchical medical policy in the current medical treatment process; this finding is similar to that of Buchner et al. [36]. For common diseases, most patients chose basic healthcare facilities and primary hospitals, which greatly contributed to the increase in the annual total number of consultations in these facilities, as indicated in our interviews. “During these years, the annual total number of consultations in my hospitals has gradually increased. Our patients are mainly patients with common diseases and those recovering from diseases” (R 6, female administrator from a primary hospital); “In my opinion, with implementation of the hierarchical medical policy, patients with common diseases are more willing to choose the nearest community healthcare service centers and basic healthcare centers for treatment” (R 8, male administrator from a basic healthcare center). In our opinion, this desirable change in patient medical behavior is attributable to awareness campaigns and effective implementation of the hierarchical medical policy and to policy measures requiring lower treatment costs and higher reimbursement ratios for medical insurance in lower-level hospitals.

Nevertheless, policymakers should note that most patients with other diseases still prefer high-level hospitals. Those with severe and incurable diseases mostly chose tertiary hospitals, which is consistent with the hierarchical medical policy objectives. For chronic diseases, most patients chose secondary and tertiary hospitals, contrary to the recommendation that “patients with chronic diseases should first go to low-level hospitals for treatment, then decide whether to be transferred to high-level hospitals, in accordance with their condition”. During recovery, patients are encouraged to receive healthcare services from primary hospitals and basic healthcare centers. Although the numbers of such patients are more balanced among the different hospital types, high-level hospitals remain the facilities of choice.

Patient choices and behaviors are driven by a number of factors [37], including hospital reputation, treatment quality, insurance plan, Internet equipment, and so on. They influence patients’ choice of hospitals to varying degrees [38,39,40], according to the research results, “level of physician diagnosis and treatment”, “severe condition of disease”, and “advanced level of medical equipment and technology”. To ensure that “acute and chronic diseases are treated by different facilities” (a goal of the hierarchical medical policy), the municipal government of Beijing needs to strengthen policy publicity and improve infrastructure and service quality in primary hospitals and basic healthcare centers.

Although the implementation of the hierarchical medical policy has not completely changed patient preferences and treatment-seeking behaviors, most respondents reported feeling satisfied with the progress in policy implementation and the phased results achieved. However, after further analysis, we found that patients were less satisfied with some aspects of the hierarchical medical policy. For example, “family doctors signing rate increases, the team of general practitioners expands” was identified as the reform measure that least met their expectations, which suggests a gap between the supply and demand for family doctors/general practitioners. In China, general practitioners mainly work in primary hospitals and basic healthcare centers. The shortage and slow increase in the number of general practitioners partly explains why many patients continue to seek treatment in high-level hospitals. Therefore, the training and provision of family doctors/general practitioners is an urgent need for the next implementation stage of the hierarchical medical policy.

### 4.3. Effects on Hospital Management

The management of Beijing public hospitals has become more modernized and rationalized after the implementation of the hierarchical medical policy. With respect to the hospital fee structure, the drug cost ratio of outpatients and inpatients in all hospital types has been decreasing, which indicates that hospitals at all levels are actively adjusting the structure and management of medical fees to respond to reform efforts to “return hospital functions to serving patients instead of selling medicines”. Our interviews confirmed this trend. “The drug cost ratio of outpatients to inpatients is a hospital assessment index closely related to the hierarchical medical policy, and the value of this index has been decreasing year by year” (R 1, male administrator from a tertiary hospital). “During the process of medical reform, we removed medicine markups and canceled registration fees and consultation fees, instead of adding medical service fees” (R 4, male administrator from a secondary hospital). Another hypothesis is that the use of artificial intelligence systems in healthcare could also help reduce the costs, which is consistent with Oliva et al.’s findings [41]. However, we noticed that the drug cost ratio of outpatients unexpectedly increased in 2018 in primary hospitals, which was the only increase in a relevant indicator. Our interviews revealed the reason for this. “We have a high proportion of outpatients with common and minor illnesses, and drug cost is an important part of the medical expenses of these patients. Also, standard medical service fees are lower in primary hospitals than in secondary and tertiary hospitals” (R 5, male administrator from a primary hospital). “A large number of patients choose to seek treatment in tertiary hospitals and then go to nearby primary hospitals to receive drugs” (R 6, female administrator, from a primary hospital).

With respect to hospital functions, the functional positioning of the different hospital types became more targeted. Tertiary hospitals are now more focused on providing inpatient services, which is reflected in the present results as a proportional increase in the usage rate of beds and a decrease in the average days of hospitalization for discharged patients. After the implementation of the hierarchical medical policy, some potential outpatients in tertiary hospitals were diverted to other hospital types, as confirmed in our study. Therefore, tertiary hospitals must expand inpatient operations to meet the policy goals and their operational needs. Thus, tertiary hospitals, on the one hand, increased the number and usage rate of beds. On the other hand, decreased average days of hospitalization for discharged patients allowed them to accept more inpatients. These measures promoted the implicit goals of the hierarchical medical policy but might increase the workload and job stress of healthcare workers in tertiary hospitals, as suggested in our interviews. “In my opinion, the development of the hospitalization business in tertiary hospitals is a policy implementation behavior, as well as a market competition behavior. Business transformation inevitably causes some confusion and stress among healthcare workers” (R 1, male administrator from a tertiary hospital). “Hierarchical medical policies have a great impact on tertiary hospitals, and we need to make adjustments in business, processes, personnel, and other aspects, which is a serious challenge for hospital managers and staff” (R 2, female administrator from a tertiary hospital).

### 4.4. Policy Prospects

The hierarchical medical policy is a signpost of China’s medical reform and has attracted great attention there and abroad. Beijing started implementing the hierarchical medical policy in 2015 and was expected to provide experience and insights for other cities. On the whole, the effects have been significant, but challenges and obstacles remain for the future implementation of the policy. Therefore, we propose the following policy suggestions.

Firstly, the Beijing government needs to further plan and publicize the hierarchical medical policy through the Internet and other ways, especially for less-educated citizens [42], implement policy by guiding hospitals and patients through economic incentives and systematically evaluate the effectiveness of policy implementation. Supporting rules and regulations should be developed, and key tasks need to be arranged for the refinement of the hierarchical medical policy in Beijing. This would involve further clarifying the functional positioning and business scope of hospitals, identifying standards and processes for patient referral, and enriching healthcare services for chronic disease in primary hospitals and basic healthcare centers. Publicity and use platforms or channels such as official websites, Weibo, and WeChat should be augmented to increase public understanding of the hierarchical medical policy. Hospitals and patients should be guided to implement the policy through their economic behavior by further improving diagnosis-related groups [43,44], thus ensuring that high-level hospitals receive patients with severe and acute diseases and low-level hospitals receive patients with less severe diseases. In addition, we suggest that patients with chronic and common diseases be reimbursed for their medical expenses only when they receive their initial diagnosis and treatment at primary medical institutions. In addition, the hierarchical medical policy should be integrated with the development of a modern hospital management system and performance evaluation, and a scientific evaluation system should be developed.

Secondly, public hospitals need to change their thinking, promote medical friendships, vigorously train general practitioners, and improve the signing rate of family doctors. Public hospitals should actively change their thinking and combine the hierarchical medical policy with hospital development strategies. For high-level hospitals, the implementation of the hierarchical medical policy diverts their patients, which could lead to resistance or passivity to reform in some hospitals and, ultimately, to their closure. Therefore, public hospitals need to modify their thinking, adjust their development strategy and functional orientation, and actively complete the phased key tasks of the hierarchical medical policy [16]. Medical partnerships should be encouraged to allow high-level hospitals to concentrate and optimize medical services, improve efficiency in the allocation of medical resources, and improve care and service capacity in lower-level hospitals. Under the premise of cyber security, using artificial intelligence to assist the hospital work to reduce costs [41]. In addition, high-level hospitals in medical friendships could prioritize patients transferred from low-level hospitals. To assist in training general practitioners, high-level hospitals should provide expert consultation and technical guidance to lower-level hospitals. In addition, training in family hospitals should be strengthened to encourage more doctors to become family doctors and provide door-to-door services for contracted patients.

Thirdly, healthcare workers should actively respond to the changes and pressures of reform, strengthen exchanges and cooperation, and improve professional skills and quality of service. On the one hand, in the face of work changes and job stress caused by the hierarchical medical policy, healthcare workers need to adjust their thinking and take appropriate measures to relieve job stress. On the other hand, healthcare workers at high-level hospitals should strengthen communication and cooperation with healthcare workers in low-level hospitals by providing regular diagnostic and treatment services and professional training, thereby improving professional skills and quality of service.

Lastly, patients need to adjust their understanding of low-level hospitals, understand the functional positioning of the different hospital types [45], and select the hospital appropriate for the nature and severity of their illness. In addition, they should discuss the hierarchical medical policy with family and friends, thereby broadening the reach of the policy.

## 5. Conclusions

We reviewed the progress of the hierarchical medical policy in Beijing, used statistical, questionnaire, and interview data to analyze its effect on healthcare workers, patients, and public hospitals, and examined future modifications of the policy. Policy implementation alleviated difficulties in obtaining medical treatment, balanced the workloads of healthcare workers in all hospital types, and changed the health-seeking behavior of patients. However, obstacles remain for the implementation process. The psychological welfare of healthcare workers was not carefully considered as part of the reform process, some healthcare services remain expensive, and the development level and service capacity of primary hospitals need further improvement. Therefore, the Beijing municipal government, public hospitals, and healthcare workers must continue to make positive changes in order to achieve the future development goals of the hierarchical medical policy.

## Figures and Tables

**Table 1:** Annual total number of patients and discharged patients of different level public hospitals in Beijing from 2009 to 2018 (10,000 person-times).

Variable	Hospitals	2009	2010	2011	2012	2013	2014	2015	2016	2017	2018
Annual total number of consultations	Tertiary	4439.3	4946.1	5554.1	8072.3	9806.2	11,058.2	11,974.9	12,525.5	11,385.6	11,318.8
	Secondary	3042.3	3393.9	3755.7	3991.3	3579.4	3385.3	3082.6	3346.2	3203.6	3267.9
	Primary	794.9	876.6	1000.2	1067.4	1143.5	1125.9	1154.8	1268.5	1365.4	1470.1
Annual total number of discharged patients	Tertiary	92.5	102.1	113.1	177.1	212.4	243.2	266.8	286.2	300.4	321.3
	Secondary	53.5	55.8	59.9	63.7	50.7	49.9	44.7	52.7	53	52.9
	Primary	9	10.2	12.6	12	12.2	12.6	13.5	14.5	14	15.3

**Table 2:** Patients for every doctor per day of different level public hospitals in Beijing from 2009 to 2018 (Person-times).

Variable	Hospitals	2009	2010	2011	2012	2013	2014	2015	2016	2017	2018
Consultations for every doctor per day	Tertiary	8.84	9.4	10.1	11.55	12.4	12.47	11.94	11.69	10.22	9.74
	Secondary	8.88	9.5	10.1	10.49	10.05	10.03	9.46	10.28	9.65	9.65
	Primary	10.01	9.7	9.9	10.47	11.78	9.59	10.06	10.23	10.21	11.16

**Table 3:** Inpatient bed days responsible for every doctor per day of different level public hospitals in Beijing from 2009 to 2018 (Day).

Variable	Hospitals	2009	2010	2011	2012	2013	2014	2015	2016	2017	2018
Inpatient Bed Days Responsible for Every Doctor Per Day	Tertiary	1.75	1.8	1.7	1.81	1.79	1.73	1.64	1.61	1.61	1.61
	Secondary	1.68	1.7	1.6	1.47	1.33	1.38	1.29	1.38	1.38	1.36
	Primary	0.81	0.7	0.7	0.67	0.65	0.6	0.62	0.59	0.49	0.48

**Table 4:** Healthcare workers’ subjective perception measurement statistics table.

Variable	Item	Mean	SD
Job satisfaction	My job is very pleasant.	3.66	0.91
	My job is very worthwhile.	3.98	0.85
	My job is better than most.	3.65	0.95
	I sometimes feel my job is a waste of time.	3.43	1.21
	I am very content with my job.	3.70	0.92
	This job is worse than most.	3.54	1.16
Challenge stress	The number of projects and or assignments I have.	3.38	0.84
	The amount of time I spend at work.	3.44	0.86
	The volume of work that must be accomplished in the allotted time.	3.41	0.87
	Time pressures I experience.	3.39	0.86
	The amount of responsibility I have.	3.47	0.88
	The scope of responsibility my position entails.	3.41	0.87
Hindrance stress	The degree to which politics rather than performance affects organizational decisions.	2.70	1.14
	The inability to clearly understand what is expected of me on the job.	2.43	1.12
	The amount of red tape I need to go through to get my job done.	3.09	1.00
	The lack of job security I have.	2.92	1.12
	The degree to which my career seems stalled.	2.96	1.10
	Medical incidents often happen.	3.02	1.21
Distributive justice	My work arrangement is reasonable.	3.61	0.94
	I get paid fairly.	3.36	1.03
	My workload is fair.	3.44	0.99
	Overall, I was paid fairly.	3.31	1.05
	My job responsibilities are fair.	3.50	0.97
Work performance	Quality of your performance.	4.01	0.71
	Your productivity on the job.	4.05	0.75
	How do you evaluate the performance of your peers at their jobs compared with yourself doing the same kind of work?	4.00	0.73
	How do you evaluate the performance of yourself at your job compared with your peers doing the same kind of work?	4.03	0.76

**Table 5:** Subjective perception ANOVA test of healthcare workers of hospitals at all levels and basic healthcare institutions.

Variable	Institutions	Mean	Mean’s 95% Confidence Interval		F	Saliency
			Lower Limit	Higher Limit		
Job satisfaction	Tertiary	3.7398	3.6469	3.8327	10.812	0.000
	Secondary	3.7148	3.6293	3.8004		
	Primary	3.5278	3.4169	3.6386		
	Basic	3.0000	2.7985	3.2015		
Challenge stress	Tertiary	3.5578	3.4634	3.6523	4.682	0.003
	Secondary	3.3001	3.1974	3.4029		
	Primary	3.3986	3.2942	3.5031		
	Basic	3.4928	3.1694	3.8162		
Hindrance stress	Tertiary	2.8155	2.7067	2.9243	2.303	0.076
	Secondary	2.7878	2.6601	2.9154		
	Primary	3.0167	2.9102	3.1232		
	Basic	3.0217	2.6540	3.3894		
Distributive justice	Tertiary	3.3633	3.2492	3.4774	6.297	0.000
	Secondary	3.6117	3.4956	3.7278		
	Primary	3.2233	3.0744	3.3723		
	Basic	3.4174	3.0755	3.7593		
Work performance	Tertiary	4.0434	3.9610	4.1257	8.288	0.000
	Secondary	4.1309	4.0402	4.2215		
	Primary	3.8313	3.7339	3.9286		
	Basic	3.6739	3.4745	3.8733		

Notes: tertiary: tertiary hospitals, secondary: secondary hospitals; primary: primary hospitals; Basic: basic healthcare institutions.

**Table 6:** Statistics on the understanding of the hierarchical medical policy.

Variable	Degree	Frequency	Percentage (%)
I know the hierarchical medical policy very well	Strongly agree	95	17.7
	More agree	175	32.6
	Common	147	27.4
	Disagree	78	14.6
	Strongly disagree	41	7.6

**Table 7:** Statistics of medical choices for different types of diseases.

Disease Type	Tertiary Hospital	Secondary Hospital	Primary Hospital	Basic Healthcare Institutions
Common diseases (such as cold, fever, etc.)	88	70	96	282
Acute diseases (such as appendicitis, acute gastroenteritis, etc.)	292	156	81	7
Chronic diseases (such as diabetes, hypertension, heart disease, chronic stomach disease, etc.)	271	151	95	19
Severe diseases (such as a tumor, uremia, acute stroke, etc.)	389	124	22	1
Incurable diseases (such as acromegaly, hemophilia, phenylketonuria, thalassemia, icing, etc.)	482	36	16	2
Recovery from diseases (such as massage, physiotherapy, traction, etc.)	165	149	108	114
Total	1687	686	418	425

**Table 8:** Statistics on the importance of factors affecting medical behavior.

Question	Item	Mean	SD
Factors affecting your choice of healthcare institutions	Severe conditions of diseases	4.48	0.83
	Medical expenses	3.95	1.02
	Convenience and reimbursement extent of medical reimbursement	3.98	1.02
	The level of the doctor’s diagnosis and treatment	4.48	0.84
	Advanced level of medical equipment and technology	4.37	0.82
	Distance to the hospital	3.92	0.92
	Service attitude of healthcare workers and hygienic environment	4.09	0.87
	Information convenience during medical treatment	3.99	0.88

**Table 9:** Statistics on the satisfaction of the implementation measures of the hierarchical medical policy.

Variable	Item	Mean	SD
Satisfaction of implementation measures of hierarchical medical policy	The construction of the hierarchical medical system is gradually improved	3.63	0.98
	High-quality resources in the medical union circulate up and down	3.57	1.02
	Government, hospitals, etc., guide people to seek medical treatment reasonably	3.62	1.04
	Pilot work for the classification and treatment of chronic diseases is well underway	3.56	1.05
	“Physician training” and “Physician support” to improve the service capabilities of primary medical institutions	3.68	0.94
	Regulatory role of medical insurance policies has been strengthened, such as setting different reimbursement ratios	3.71	1.00
	Family doctor’s signing rate increases, the general practitioner’s team expands	3.55	1.11
	Informatization construction is gradually strengthened, and the patient’s medical treatment is more convenient	3.77	0.96

**Table 10:** Statistics on the overall satisfaction of the hierarchical medical policy.

Variable	Degree	Frequency	Percentage (%)
Your satisfaction with the overall implementation of the hierarchical medical policy in your area is (Overall satisfaction)	Strongly satisfied	94	17.5
	More satisfied	172	32.1
	Common	220	41.0
	Unsatisfied	40	7.5
	Strongly unsatisfied	10	1.9

**Table 11:** Per capita cost of outpatients and inpatients of different level public hospitals in Beijing from 2009 to 2018 (Yuan).

Variable	Hospitals	2009	2010	2011	2012	2013	2014	2015	2016	2017	2018
Per capita cost of outpatients	Tertiary	394.4	429.3	447.2	456.9	447.6	448.5	467.6	480.2	539.1	569.5
	Secondary	224	246.1	263	287.4	305.8	327.5	346.1	356.9	377.3	368.2
	Primary	155.7	203.3	168.1	195.8	219.2	169.3	183.6	181.5	242.2	331.2
Per capita cost of inpatients	Tertiary	18,347.8	19,386.2	19,764.2	20,557.3	19,510.8	19,499.9	20,706	21,234.2	22,321	23,097.3
	Secondary	9384.7	10,502.4	11,011.7	11,748.2	13,233.7	14,339.8	14,895.9	15,155.2	15,606.8	15,813.4
	Primary	5299.5	5039.1	5360.8	6797.9	5603.5	3988.4	4120	7134.1	13,249.2	10,135.4

**Table 12:** Drug cost ratio of outpatients and inpatients of different level public hospitals in Beijing from 2009 to 2018 (%).

Variable	Hospitals	2009	2010	2011	2012	2013	2014	2015	2016	2017	2018
Drug cost ratio of outpatients	Tertiary	63.07	63.72	62.76	62.18	59.01	57.94	56.05	54.71	46.96	43.46
	Secondary	68.15	69.51	68.15	67.66	68.39	68.51	68.05	66.18	59.02	51.92
	Primary	71.86	67.3	66.47	68.32	64.35	77.86	81.7	74.21	65.39	78.14
Drug cost ratio of inpatients	Tertiary	34.64	35.56	32.36	30.99	29.16	28.19	27.06	26.83	22.53	21.44
	Secondary	43.63	44.19	40.94	41.23	39.79	38.79	39.26	37.68	31.18	27.92
	Primary	39.64	47	48.76	47.06	23.97	54.94	56.16	46.21	27.29	15.47

**Table 13:** The usage rate of prepared beds and available beds of different level public hospitals in Beijing from 2009 to 2018 (%).

Variable	Hospitals	2009	2010	2011	2012	2013	2014	2015	2016	2017	2018
Usage Rate of Prepared Beds	Tertiary	86.75	90.03	92.55	88.74	100.67	88.05	85.33	84.77	85.03	87.31
	Secondary	86.58	89.6	81.89	76.66	75.41	72.43	68.62	69.34	71.97	69.92
	Primary	54.75	53.02	54.27	51.65	50.05	47.12	45.76	46.88	43.1	46.85
Usage Rate of Available Beds	Tertiary	93.1	93.87	94.89	94.24	89.41	94.27	91.87	92.28	92.02	93.87
	Secondary	85.17	85.73	84.06	83.38	80.55	77.7	73.53	76.75	77.53	75.43
	Primary	61.39	59.25	59.9	57.63	54.04	50.42	46.8	48.13	46.1	47.62

Notes: Prepared Beds: number of beds approved by the Health and Family Planning Administration; Available Beds: fixed number of beds at the end of the year.

**Table 14:** The average hospitalization day of discharged patients of different level public hospitals in Beijing from 2009 to 2018 (Day).

Variable	Hospitals	2009	2010	2011	2012	2013	2014	2015	2016	2017	2018
Average Hospitalization Day of Discharged Patients	Tertiary	12.7	12.3	11.5	10.9	10.4	9.8	9.4	9.1	8.9	8.8
	Secondary	13.9	14.4	15.9	11.8	12.1	12.4	12.6	11.3	10.2	11
	Primary	19	16.2	14.9	14.3	16.5	11.6	12.3	12.6	13.5	12.6

## Data Availability

Relevant data can be found through http://www.phic.org.cn/tjsj/wstjjb/ and http://www.bjstats.gov.cn/tjsj/.
